# A Porphodimethene Chemical Inhibitor of Uroporphyrinogen Decarboxylase

**DOI:** 10.1371/journal.pone.0089889

**Published:** 2014-02-25

**Authors:** Kenneth W. Yip, Zhan Zhang, Noriko Sakemura-Nakatsugawa, Jui-Wen Huang, Nhu Mai Vu, Yi-Kun Chiang, Chih-Lung Lin, Jennifer Y. Y. Kwan, Shijun Yue, Yulia Jitkova, Terence To, Payam Zahedi, Emil F. Pai, Aaron D. Schimmer, Jonathan F. Lovell, Jonathan L. Sessler, Fei-Fei Liu

**Affiliations:** 1 Ontario Cancer Institute/Campbell Family Cancer Research Institute, University Health Network (UHN), Toronto, Ontario, Canada; 2 Department of Chemistry, Institute for Cellular and Molecular Biology, the University of Texas at Austin, Austin, Texas, United States of America; 3 Biomedical Technology and Device Research Labs, Industrial Technology Research Institute, Hsin-chu, Taiwan; 4 Department of Medical Biophysics, University of Toronto, Toronto, Ontario, Canada; 5 Department of Biochemistry, University of Toronto, Ontario, Canada; 6 Department of Molecular Genetics; University of Toronto, Ontario, Canada; 7 Department of Biomedical Engineering, University at Buffalo, State University of New York, Buffalo, New York, United States of America; 8 Department of Radiation Oncology, Princess Margaret Cancer Centre, UHN, Toronto, Ontario, Canada; 9 Department of Radiation Oncology, University of Toronto, Toronto, Ontario, Canada; Oak Ridge National Laboratory, United States of America

## Abstract

Uroporphyrinogen decarboxylase (UROD) catalyzes the conversion of uroporphyrinogen to coproporphyrinogen during heme biosynthesis. This enzyme was recently identified as a potential anticancer target; its inhibition leads to an increase in reactive oxygen species, likely mediated by the Fenton reaction, thereby decreasing cancer cell viability and working in cooperation with radiation and/or cisplatin. Because there is no known chemical UROD inhibitor suitable for use in translational studies, we aimed to design, synthesize, and characterize such a compound. Initial *in silico*-based design and docking analyses identified a potential porphyrin analogue that was subsequently synthesized. This species, a porphodimethene (named PI-16), was found to inhibit UROD in an enzymatic assay (IC_50_ = 9.9 µM), but did not affect porphobilinogen deaminase (at 62.5 µM), thereby exhibiting specificity. In cellular assays, PI-16 reduced the viability of FaDu and ME-180 cancer cells with half maximal effective concentrations of 22.7 µM and 26.9 µM, respectively, and only minimally affected normal oral epithelial (NOE) cells. PI-16 also combined effectively with radiation and cisplatin, with potent synergy being observed in the case of cisplatin in FaDu cells (Chou-Talalay combination index <1). This work presents the first known synthetic UROD inhibitor, and sets the foundation for the design, synthesis, and characterization of higher affinity and more effective UROD inhibitors.

## Introduction

Our group recently identified uroporphyrinogen decarboxylase (UROD) as a potential anticancer target *via* a high throughput siRNA screen [1], [2]. Subsequent work confirmed that: 1. siRNAs for UROD (siUROD) reduced cancer cell viability, particularly in head and neck cancer cells [1]; 2. siUROD minimally affected normal oral epithelial (NOE) and pharyngeal cells [1]; 3. siUROD promoted radiosensitivity even under conditions of hypoxia [1]; 4. siUROD sensitized cells to various chemotherapies [1]; 5. UROD was significantly upregulated in head and neck squamous cell carcinoma (HNSCC) patient samples [1]; and 6. UROD may be a clinical predictive marker for response to radiation therapy, in that patients with lower levels of pretreatment UROD experienced an improved disease-free survival [1]. The discovery and characterization of UROD inhibitors is an important translational opportunity in cancer because such chemicals may provide a potential strategy for single-agent efficacy, radiosensitization, and/or chemosensitization in a broad range of human malignancies.

UROD is the 5^th^ enzyme in the heme biosynthesis pathway, catalyzing the conversion of uroporphyrinogen to coproporphyrinogen, porphyrin molecules (macrocycles with tetrapyrroles interconnected *via* methine bridges) containing four propionic groups. Heme prosthetic groups all contain an iron atom (Fe) at the center of a porphyrin, and although heme and heme-containing proteins have diverse biological functions, major roles include regulating iron and the storage, control, and manipulation of molecular oxygen and related species. They can also serve as either a “source” or “sink” for electrons during redox reactions [3]. Sudden perturbation of iron homeostasis by UROD inhibition in cancer cells is thought to be at least partially responsible for the effectiveness of UROD as an anticancer target [1]. Consistent with this suggestion is the finding that UROD inhibition by siUROD reduces heme production, thereby increasing the amount of free ferrous (Fe^+2^) and ferric (Fe^+3^) iron, and resulting in elevated reactive oxygen species (ROS) concentrations *via* the Fenton reaction (Fe^+2^+ H_2_O_2_ → Fe^+3^+ OH*+OH^−^) [1], [2], [4]. ROS, such as the highly reactive hydroxyl radical (OH*), cause direct damage to many cellular structures, and provide a link between UROD inhibition, radiotherapy, and many chemotherapies [5]–[7]. Given the extensive level of metabolic dysregulation associated with cancer cells (reviewed in [8]), it is not surprising that iron regulation and anti-oxidant response mechanisms can be exploited for cancer therapy (reviewed in [9], [10]).

Humans deficient in UROD present with porphyria cutanea tarda (PCT), a condition characterized by light-sensitive dermatitis, excretion of excess uroporphyrins, and associated hepatic porphyrin accumulation [11]. *UROD* mutation homozygosity or compound heterozygosity causes the rare hepatoerythropoietic porphyria (HEP), which presents with pink/red-colored urine, bullous skin lesions on light-exposed areas of the skin, hypertrichosis, skin fragility, and disfiguring skin thickening/scarring [12], [13]. It is therefore anticipated that UROD inhibition may be tolerated for cancer therapy. This conjecture, however, requires careful study.

Although UROD is a potential anticancer target and crystal structures of human UROD have been elucidated [14]–[16], there exists no known chemical UROD inhibitor. An endogenous porphomethene inhibitor has been previously suggested [17]. However, the existence of the small molecule in question is controversial due to an inability to observe it directly *via* high-performance liquid chromatography (HPLC)/electrospray ionization tandem mass spectrometry and its expected chemical instability [17], [18]. The current study presents the first functional UROD inhibitor, a synthetic tetrapyrrole that was rationally designed using structure-based *in silico* approaches before being synthesized and characterized. This work provides an experimental basis for the design and preparation of more potent and bioavailable molecules that could serve as chemical probes or potential therapeutics.

## Materials and Methods

### Design and *In Silico* Docking

**Figure 1:**
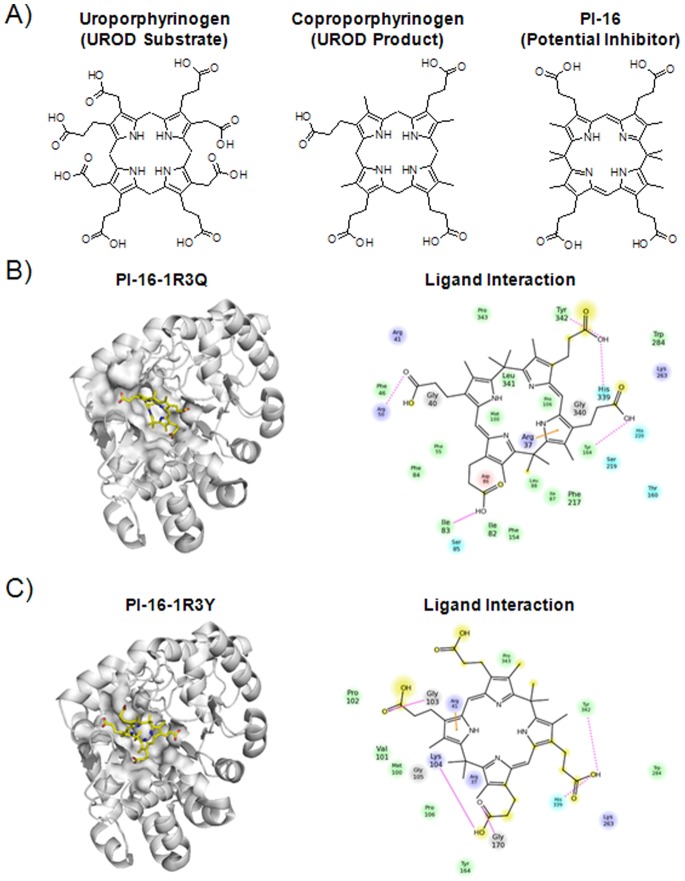
Docking of PI-16, a potential UROD inhibitor. A) Chemical structures of uroporphyrinogen, coproporphyrinogen, and PI-16. B) Sample PI-16-1R3Q UROD docking (*left*), with chemical interactions and residues 4Å from the ligand center (*right; dotted lines indicate hydrogen bonds with side chains, solid pink lines indicate backbone hydrogen bonds; solid orange lines indicate π-cation).* C) Sample PI-16-1R3Y UROD docking.

Various potential target compounds were drawn using ChemDraw (Perkin Elmer, Waltham, Massachusetts) based on their similarity to coproporphyrinogen, uroporphyrinogen, and a previously suggested endogenous inhibitor (Figure 1A, Figure S1) [17]. PI-16, the only chemically stable target compound within the set of proposed inhibitors, was docked to wildtype human UROD crystal structures (PDB codes 1R3Q and 1R3Y, [15]) using Schrödinger Suite and Glide software (Schrödinger, Munich, Germany) [19]–[21]. The multistep Schrödinger protein preparation wizard tool (PPrep) was used. Protein minimization used the OPLS-2005 force field with the Polak-Ribiere Conjugate Gradient (PRCG) algorithm. The LigPrep module was used for ligand preparation. All ligands were minimized using the OPLS-2005 force fields with the appropriate default settings. At least 10 docking poses and the corresponding scores were evaluated in both the standard precision and extra precision mode (Glide XP) for each potential target. Coproporphyrin (the oxidized product of normal UROD catalysis) was used as a control because it is the only known ligand that has been co-crystallized with UROD.

### Chemical Synthesis and NMR Spectra

#### General

All reagents and solvents were purchased from commercial suppliers and used without further purification. Low resolution mass spectra (MS) and high-resolution mass spectra (HRMS) were taken on an Ion Spec Fourier Transform mass spectrometer. Proton and ^13^C NMR spectra were recorded using a Varian 400 spectrometer (Palo Alto, California) and chemical shifts were reported in ppm using tetramethylsilane (TMS) as the reference standard.

#### Porphodimethene compound 6

Dipyrromethane dicarboxylic acid (0.87 g, 2 mmol) was dissolved in 2 mL trifluoroacetic acid (TFA) at room temperature, stirred for 20 min, chilled to −10°C, and treated with dimethoxypropane (2 mmol). The reaction mixture was kept at this temperature for 30 min and subsequently transferred to a flask containing 10 mL dichloromethane (DCM) at −20°C. To quench the reaction, ammonium hydroxide and water were added drop-wise until the pH of the aqueous phase reached 7. The organic layer was then collected and dried. 2,3-Dichloro-5,6-dicyano-1,4-benzoquinone (DDQ, 2.2 mmol) and 5 mL methanol were added to the solution, which was then allowed to stir overnight. Under these conditions, the product was found to precipitate. Yield: 73%. The free-base form of **6** does not dissolve well in organic solvents. However, its protonated form dissolves very well in chloroform-*d* (CDCl_3_). ^1^H NMR (400 MHz, CDCl_3_ containing a small amount of TFA-*d*) *δ = *12.04 (s, 4 pyrrole NH), 7.93 (s, 2 *meso*-CH), 3.69 (s, 4 CH_3_), 3.02-2.99 (t, *J = *7.6 Hz, 4 CH_2_), 2.57-2.53 (t, *J = *7.6 Hz, 4 CH_2_), 2.24 (s, 4 CH_3_), 1.86 (s, 4 CH_3_). ^13^C NMR (100 MHz, CDCl_3_) *δ* = 173.4, 158.2, 145.4, 130.6, 128.8, 125.7, 52.1, 42.6, 34.5, 27.6, 19.9, 12.2. MS (ESI) *m/z* 769.4 ([M+1]^+^). HRMS (ESI, [M+1]^+^) Calcd for C_44_H_57_N_4_O_8_; 769.41949. Found: 769.41709 (Figure S2).

#### Porphodimethene inhibitor 16 (PI-16)

A suspended methanol solution (20 mL) of 0.38 g (0.5 mmol) compound **6** was treated with NaOH (0.4 g in 20 mL water) at room temperature. The mixture was heated at reflux overnight, leading to a homogeneous brown solution. After the solution was allowed to cool to room temperature, concentrated HCl was added drop-wise until precipitation of a red/purple precipitate was complete. The solid was collected by filtration and washed with water. Yield: 65%. As true for compound **6**, the free-base form of PI-16 does not dissolve well in organic solvents. Although protonated PI-16 dissolves very well in methanol, DMSO, and TFA, the peaks in the ^1^H NMR spectrum are broad. Moreover, no reliable ^13^C NMR spectrum could be obtained, even when the sample was scanned overnight. ^1^H NMR (400 MHz, TFA-*d*) *δ = *7.94 (s, 2 *meso*-CH), 3.16 (br, 4 CH_2_), 2.76 (br, 4 CH_2_), 2.01 (br, 4 CH_3_), 1.78 (br, 4 CH_3_). MS (ESI) *m/z* 713.6 ([M+1]^+^). HRMS (ESI, [M+1]^+^) Calcd for C_40_H_49_N_4_O_8_; 713.3550. Found: 713.3754 (Figure S2C).

PI-16 was dissolved in dimethyl sulfoxide (DMSO, Sigma-Aldrich, St. Louis, Missouri) so that the final concentrations were less than 5% for the enzyme assay studies and less than 0.3% for the cellular assays.

### Recombinant Proteins

A plasmid containing *Homo sapiens* porphobilinogen deaminase (PBGD; gene accession NM_000190) cDNA with a 5′-histidine tag was obtained from Qiagen (Hilden, Germany) and transferred into *Escherichia coli* BL21-RIL(DE3) Codon Plus (Agilent, Mississauga, Canada). Cells were incubated in Luria Broth with 35 mM kanamycin (Sigma-Aldrich) at 37°C (200 rpm shaking). When the culture reached an OD_600_ = 1.0, 0.25 mM IPTG (Sigma-Aldrich) was added to express PBGD for 16 hours. PBGD was then purified using a previously published method with modifications [22]. Briefly, harvested cells were lysed by sonication in lysis buffer (20 mM Tris pH 8.5, 3 mM imidazole, 1 mM TCEP, 200 µM PMSF, 0.2% Triton X-100). The cell lysate was centrifuged at 18,000×g for 15 minutes and the supernatant was heated to 60°C for 10 min. Denatured cell proteins were removed by re-centrifugation at 18000×g for 15 min. The final, clear, supernatant was passed through Ni-NTA resin (Qiagen), and the resin washed with at least 20 column volumes of washing buffer (20 mM Tris pH 8.5). PBGD was eluted using 5 column volumes of wash buffer containing 500 mM imidazole. PBGD was further purified using gel filtration chromatography (Hiload Superdex 75, GE Healthcare, Little Chalfont, United Kingdom) with an appropriately selected buffer (100 mM Tris, pH 8.5, 5 mM DDT).

*Homo sapiens* uroporphyrinogen decarboxylase (UROD; GenBank Accession BC001778) was subcloned into the pET15b vector (Addgene, Cambridge, Massachusetts) so that the gene contained a 5′-Histidine tag with a thrombin cleavage site. Valine at UROD amino acid 303 was mutated to glycine by site directed mutagenesis to match the protein sequence published by Whitby *et al* [14]. This vector was transferred into *E. coli* BL21-RIL(DE3); cells were grown in Luria Broth with 100 mM ampicillin at 37°C (200 rpm shaking). When the culture reached an OD_600_ = 1.0, 0.25 mM IPTG was added to express UROD for 16 hours. Cells were then harvested by centrifugation and the subsequent cell pellet was re-suspended in 50 mM Tris, 500 mM NaCl, pH 8.5 lysis buffer. UROD was isolated by sonication, centrifugation (18,000×g), and Ni-NTA purification. Impurities in the Ni-NTA resin were washed away with lysis buffer and UROD was eluted with lysis buffer containing 300 mM imidazole. Recombinant UROD was further purified using gel filtration chromatography with a buffer consisting of 20 mM Tris pH 8.0 and 100 mM NaCl.

### Enzyme Assays

Enzyme assays were based on previously published methods [23]. For the UROD assay, PBG (32 µM; Frontier Scientific, Logan, Utah) was incubated with recombinant PBGD (6.15 µM) in 0.1 M Tris, pH 7.65, 7.5 mM DTT at 37°C for 35 min to produce uroporphyrinogen. Uroporphyrinogen (2 µM) was then incubated with recombinant UROD (0.25 µM, 20 µL final reaction volume) and the compound subject to analysis in 50 mM KH_2_PO_4_, pH 6.8 at 37°C 1 h, followed by HCl (5 µL, 2.5 M), UV light (30 min), NaOH (6 µL, 6.5 M), and ethanol (65 µL). For the PBGD assay, PBG (32 µM) was incubated with PBGD (0.179 µM) and the compound subject to analysis in 0.1 M Tris, pH 7.65, 7.5 mM DTT at 37°C for 35 min, followed by HCl (5 µL, 2.5 M), UV light (30 min), NaOH (6 µL, 6.5 M), and ethanol (65 µL). All enzyme assay samples were diluted with 50 µL DMSO and analyzed for substrate, intermediate, and product concentrations using reverse phase high pressure liquid chromatography (RP-HPLC; C18 column, 20% to 40% ACN eluent) and fluorescence detection (405 nm).

### Cellular Assays

FaDu (human hypopharyngeal squamous carcinoma) and ME-180 (human cervical carcinoma) cells were obtained from the American Type Culture Collection (ATCC, Manassas, VA). NOE (human normal oral epithelial) cells were purchased from Celprogen (San Pedro, CA). All cell lines were cultured according to specifications in 5% CO_2_, 21% O_2_, and 95% humidity at 37°C and confirmed to be mycoplasma negative every 3 months (MycoAlert, Lonza, Basel, Switzerland).

For viability assays, cells were seeded (2000 cells/well) in 96-well plates for 24 h, after which the compounds subject to study, controls, and/or radiation were added at the indicated times, concentrations, and/or doses. ATPlite (PerkinElmer) was used to measure cell viability according to the manufacturer’s specifications.

For clonogenic assays, cells were seeded (100–1500 cells/well) in 12-well plates, and 24 h later, compounds or controls were added at the indicated concentrations. After another 24 h, the cells were irradiated where indicated. Approximately 10–12 days later, colonies were fixed in 70% ethanol, stained with 10% methylene blue, and colonies of ≥50 cells were counted.

Cellular studies used cisplatin from Mayne Pharma Canada (Kirkland, Canada) and a Gammacell 40 Extractor (dose rate 1.1 Gy/min; MDS Nordion, Ottawa, Canada) for radiation where indicated.

### Statistical Analyses

All experiments were performed at least three independent times. The mean values were then tabulated with either the standard deviation or the standard error of the mean being presented as indicated. The effect of PI-16 with radiation or with cisplatin was evaluated using the Chou-Talalay combination index (CI) method [24], [25].

## Results

### Design of a Potential UROD Inhibitor

Based on the structures of uroporphyrinogen, coproporphyrinogen, and a previously suggested endogenous inhibitor [17], various theoretical compounds were rationally designed as possible inhibitors. The previously reported endogenous inhibitor was a partially oxidized porphyrinogen [17]. Unfortunately, porphyrinogens and partially oxidized porphyrinogens that bear unsubstituted methylene group(s) at the *meso* position are exceedingly unstable. Well-known decomposition pathways include rapid oxidation to porphyrins in the presence of air. Our goal, therefore, was to develop stable, partially oxidized porphyrinogen inhibitors by direct chemical synthesis. Towards this end, the preparation of several different types of nonconjugated porphyrin derivatives was attempted. Targets included porphodimethenes, oxophlorins, and sulfur-bridged macrocycles (Figure S1). However, only PI-16 proved sufficiently stable to allow for its analysis as a potential UROD inhibitor (Figure 1A). We named this porphodimethene inhibitor PI-16, as it was the 16^th^ compound designed.

Support for the contention that PI-16 would dock effectively with UROD came from analyses of UROD crystal structures 1R3Q and 1R3Y (PDB Codes, [15]), which are the wild-type human UROD structures co-crystallized with coproporphyrin (oxidized coproporphyrinogen) I and coproporphyrin III isomer products, respectively. Notably, no known co-crystallized structure exists with uroporphyrinogen or coproporphyrinogen, likely due to the rapid action of UROD on these easy-to-oxidize substrates [26]. Likewise, no structure exists wherein uroporphyrin (the oxidized form of uroporphyrinogen) is bound to UROD. PI-16, on the other hand, was hypothesized to be chemically stable because of the presence of two quaternary carbons; similar but more simplified porphodimethenes have been well-characterized [27]. PI-16 was found to dock to 1R3Q with a Glide score of 6.98 (Root-mean-square deviation of atomic positions (RMSD) = 7.8824Å), and to 1R3Y with a Glide score of 5.16 (10.4175Å RMSD). The co-crystallized ligands were used as controls for comparison; coproporphyrin I had a Glide score of 9.44 (3.8854Å RMSD) on 1R3Q and coproporphyrin III had a Glide score of 4.94 (9.9177Å RMSD) on 1R3Y. As illustrated in Figure 1B & 1C, the major interactions between PI-16 and UROD may involve numerous hydrogen bonds and one aromatic π-π interaction. The illustrated interactions are individually described in Figure S3.

### Synthesis of a Potential UROD Inhibitor

**Figure 2:**
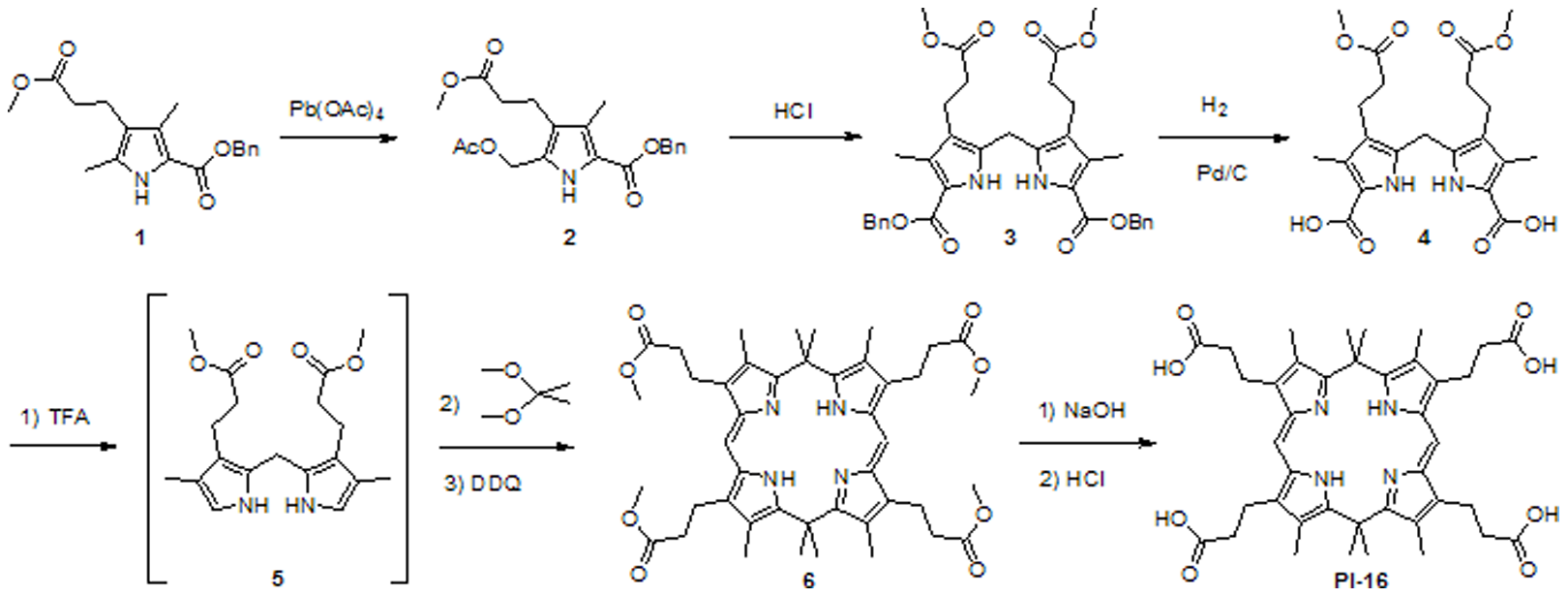
Synthesis of PI-16. PI-16 was synthesized by lead acetate-mediated oxidation of pyrrole ester to create an acetoxymethyl pyrrole ester, followed by condensation to generate a dipyrromethane diester. Subsequent hydrogenation, catalyzed by palladium on carbon (Pd/C), produced the corresponding dipyrromethane diacid, which was decarboxylated by treating with trifluoroacetic acid (TFA). This intermediate was cyclized in the presence of acetone dimethyl acetal, followed by ammonium hydroxide quenching, dichloromethane (DCM) extraction, and 2,3-dichloro-5,6-dicyanobenzoquinone (DDQ) oxidation to yield PI-16.

Dipyrromethanes are key precursors of porphyrins and porphyrin derivatives. Because the synthesis of dipyrromethane diacid (Figure 2, compound **4**) has been well-established [28], the synthetic route shown in Figure 2 was devised. Starting from the commercially available pyrrole ester (**1**), diacid **4** was prepared on a large scale *via* lead acetate-mediated oxidation, acidic condensation, and Pd-catalyzed hydrogenation. The yields of each step were consistently high. Next, α-free dipyrromethane (**5**) was generated through decarboxylation of **4** in TFA at room temperature, and the ensuing cyclization was enacted *in situ*, but at −10°C. Acetone dimethyl acetal was used as the reactant in this cyclization step. The use of unreactive acetone as a potential condensation partner was found to give rise to increased levels of polymerization as compared to cyclization. The success of the cyclization step was also discovered to depend on the reaction temperature. Further, cyclization depended on the reagent used to quench the condensation and the temperature at which the quenching was effected. In practice, acceptable yields (73%) were obtained when pre-chilled aqueous ammonium hydroxide was used to quench the reaction. In order to avoid undesired degradation of the cyclization product, the extracted organic fraction was oxidized immediately by treatment with DDQ. This led to the precipitation of the porphodimethene tetraester (**6**) from solution. After hydrolysis under basic conditions, the desired compound PI-16 was precipitated by adding HCl to the solution and then collecting the resulting solids *via* filtration. PI-16 as obtained in this way was purple in color, a finding that was taken as an indication that the compound was still in its protonated form. The precipitate was washed with a significant amount of water while on the filter as to remove excess acid. Although the NMR spectra of precursor compound **6** were characterized by sharp peaks, those of PI-16 proved very broad (Figure S2). Moreover, few discernible peaks were observed in the ^13^C NMR spectrum. Such findings are consistent with the notion that PI-16 is subject to considerable conformational motion in acidic environments. Nevertheless, the proton integration of PI-16 agreed very well with its structure. High resolution mass spectroscopic analysis provided further support for the conclusion that PI-16 was synthesized successfully and the chemical structure correctly assigned (Figure S2D).

### Biochemical Characterization of PI-16

**Figure 3:**
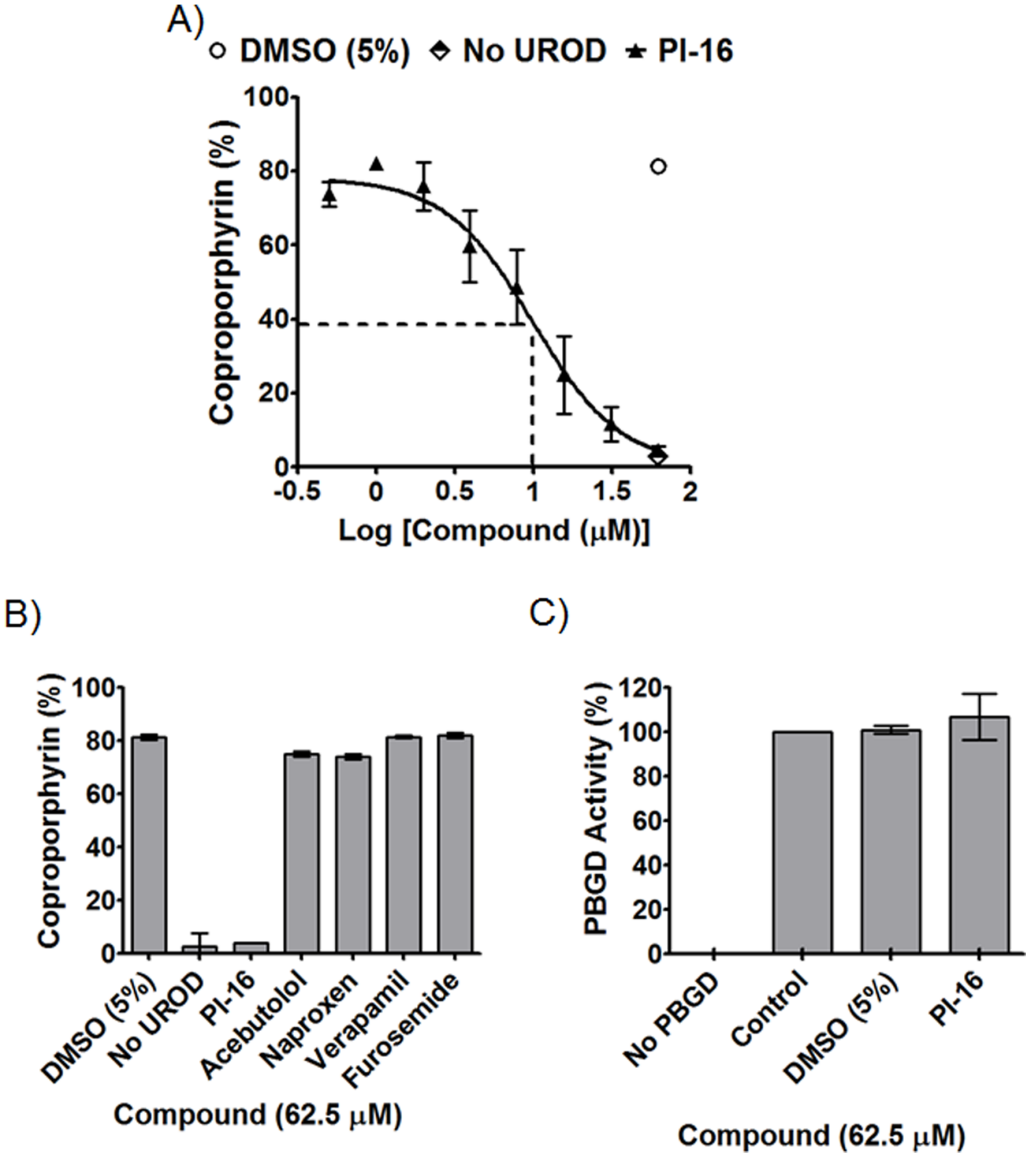
Biochemical characterization of PI-16. A) PI-16 inhibited UROD (0.25 µM UROD, 2 µM uroporphyrinogen, 37°C 1 h) with IC_50_ = 9.9 µM (*dotted line indicates half-maximal*). This enzyme assay used RP-HPLC (C18 column, 20%–40% acetonitrile elution) to measure oxidized substrate (uroporphyrin) and product (coproporphyrin) concentrations. B) Miscellaneous compounds did not inhibit UROD. C) Porphobilinogen deaminase (PBGD) was not inhibited by PI-16, suggesting specificity (0.179 µM PBGD, 32 µM uroporphyrinogen, 37°C 1 h). Reaction conditions represent Km/linear range, with mean and standard error of the mean from three independent experiments are shown.

A recombinant-UROD enzyme assay that utilizes RP-HPLC for the detection of oxidized substrates, intermediates, and products, was used to confirm PI-16-mediated inhibition of UROD. Under the conditions of these studies, UROD was inhibited with a half maximal inhibitor concentration (IC_50_) of 9.9 µM (Figure 3A). Although at first blush, this IC_50_ level may appear modest, in fact PI-16 was judged to be quite potent since 0.25 µM UROD and 2 µM uroporphyrinogen (*in vitro* Michaelis constant, or Km, concentrations) were used. Inhibitor specificity could be observed: Firstly, a panel of randomly selected compounds did not inhibit UROD (Figure 3B); secondly, PI-16 did not inhibit PBGD, another enzyme in the heme biosynthesis pathway, even when PI-16 was tested at a concentration of 62.5 µM (Figure 3C).

### Cellular Characterization of PI-16

**Figure 4:**
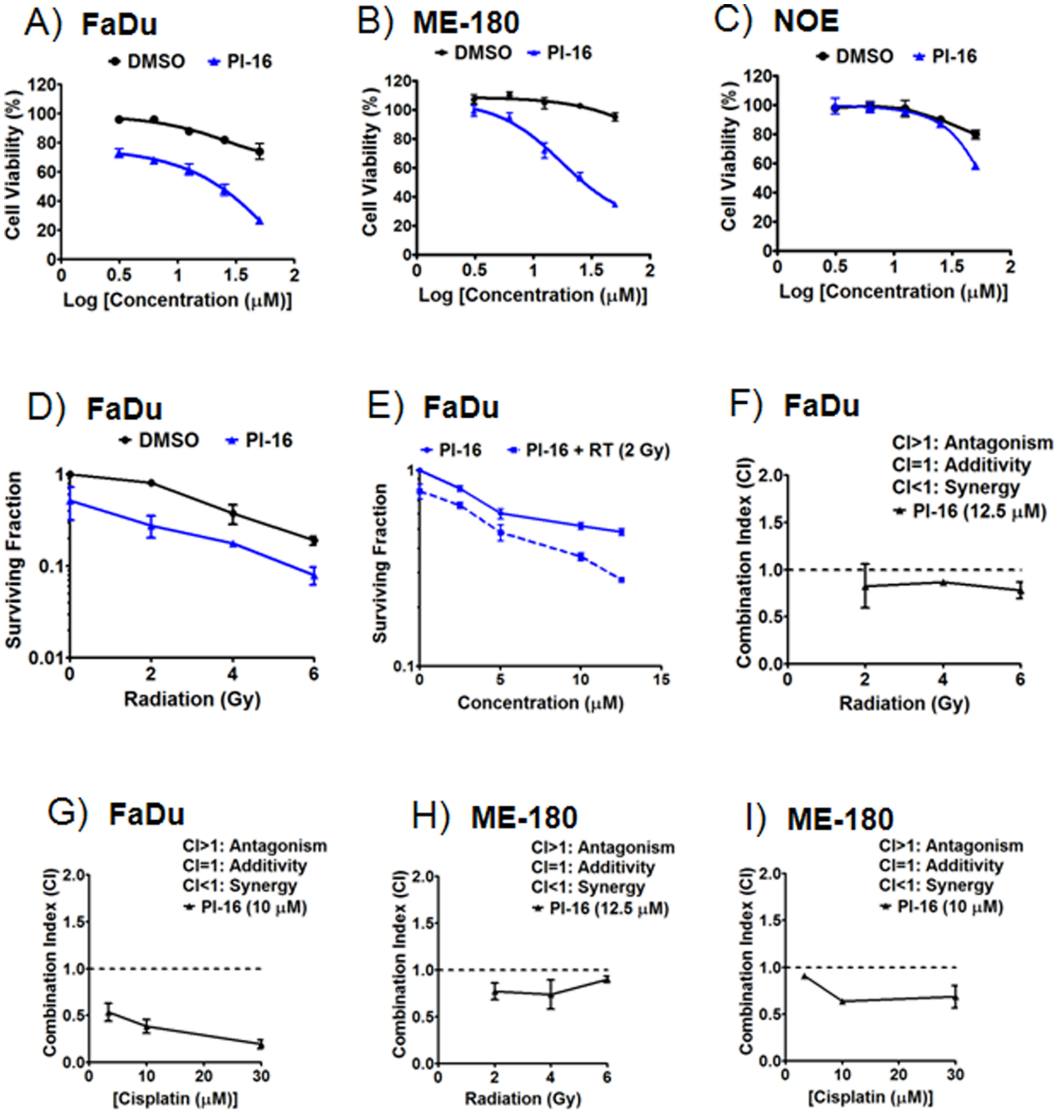
Cellular characterization of PI-16. A & B) FaDu (human hypopharyngeal carcinoma; *A*) and ME-180 (human cervix cancer; *B*) cells demonstrated a reduction in cell viability (ATPlite, PerkinElmer) when seeded for 24 h and incubated with PI-16 for 48 h. C) NOE (human normal oral epithelial) cells were minimally affected when seeded for 24 h, then incubated with PI-16 for 48 h. D & E) Clonogenic assays were performed by seeding FaDu cells, irradiating after 24 h, and then adding compounds after another 24 h. 12.5 µM and 0.1% DMSO were used in *D*, and 2 Gy radiation in *E*. F) Clonogenic assay-based Chou-Talalay combination indices [24], [25] between PI-16 and radiation in FaDu cells. G, H, I) Similar viability experiments indicate synergy between PI-16 and cisplatin in FaDu cells, and more-than-additive effects between PI-16 and radiation or cisplatin in ME-180. All experiments were performed in triplicate, with equivalent concentrations of DMSO used as controls. Mean and standard error of the mean (or standard deviation for combination indices) from three independent experiments are shown.

UROD inhibition using siUROD was previously characterized in FaDu (human hypopharyngeal squamous carcinoma), ME-180 (human cervical carcinoma), and NOE (human normal oral epithelial) cells [1]; we therefore evaluated PI-16 on the same cell lines. The compound decreased viability in FaDu and ME-180 cells (half maximal effective concentration or EC_50_ = 22.7 µM and 26.9 µM, respectively) much more effectively than in NOE cells (EC_50_>50 µM) (Figure 4A, 4B, & 4C). These observations are consistent with what was previously found with siUROD [1].

In order to assess potential effects with radiation, clonogenic assays were performed with PI-16. At-least-additive interactions were seen based on the results obtained using Chou-Talalay CI in FaDu cells (CI<1; Figure 4D, 4E, 4F; [24], [25]). Further experiments with cisplatin provided evidence that combination treatment with PI-16 was potently synergistic (Figure 4G; using a concentration of PI-16 that reduces clonogenicity by ∼50%). What appeared to be more-than-additive interactions were also be observed with PI-16 and radiation/cisplatin in ME-180 cells (Figure 4H & 4I). Collectively, the cellular effects of PI-16 are comparable to those of siUROD (e.g., CI<1). However, siUROD experiments differ from those involving PI-16 in that they require nanomolar quantities of siRNA but also a transfection reagent [1]. The potential advantages of the synthetic material (cost, ease of handling, no requirement for a transfection agent) led us to study PI-16 in further detail.

### Preliminary *In Vivo* Assessment of PI-16

Unfortunately, PI-16 proved poorly soluble in aqueous media. It was therefore not immediately suitable for systemic delivery in animal studies without formulation with an excipient. However, by using Cremophor, it proved possible to generate solutions suitable for intraperitoneal (IP) injections into mice at 40 mg/kg. Preliminary animal data indicated a small but not statistically significant increase in time-to-endpoint with this molecule combined with radiation in mice harboring FaDu cell tumors in their left gastrocnemius muscle (File S1 for methods and Figure S4). On the other hand, PI-16 did not give rise to any deleterious effects, as inferred from analyses of mouse body weight and general health (e.g., fur coat or behavior). However, these animal data must be interpreted cautiously and further work is needed with respect to the formulation and characterization of PI-16 *in vivo*.

## Discussion

The current study describes the identification, synthesis, and characterization of a novel porphodimethene UROD inhibitor, and sets the foundation for the synthesis of more potent chemicals. *In silico*, this compound docks to the UROD structures 1R3Q and 1R3Y with scores comparable to those of coproporphyrin I and III, respectively. Biochemically, PI-16 inhibits UROD (IC_50_ = 9.9 µM using 0.25 µM UROD and 2 µM uroporphyrinogen) without affecting PBGD, even at substantially higher concentrations (e.g., 62.5 µM), supporting the notion that it exhibits specificity. In cellular assays, PI-16 resembled siUROD in that it reduced the viability of FaDu (EC_50_ = 22.7 µM) and ME-180 (EC_50_ = 26.9 µM) cancer cells more than NOE (normal; EC_50_>50 µM) cells. This compound may be used effectively *in vitro* in conjunction with radiation and cisplatin. In particular, it provides potent and demonstrated synergistic behavior when used in combination with cisplatin in FaDu cells (CI<1, Figure 4G).

One major limitation to PI-16 is its poor solubility in aqueous media. Although it can be dissolved in DMSO to a certain extent, this feature severely limits work with animal models and largely precludes systemic delivery methods such as intravenous (IV) injections without optimizing a delivery formulation. IV injections are normally preferable for porphyrin-based molecules because they facilitate optimal distribution and allow for porphyrin-mediated preferential cancer cell uptake [29]–[33]. Thus, IP-administered PI-16 was anticipated to have limited effects in animal models, particularly in tumor models located outside of the peritoneal cavity. Future studies will focus on modifications that increase the *in vivo* applicability of UROD inhibitors, whether by chemical modification and/or mechanism of delivery, such as with liposomes or porphysomes [34]–[36]. These modifications will of course require biochemical and cellular re-assessment. Nevertheless, the current study provides a first proof-of-concept demonstration of a synthetic UROD inhibitor. As such, it sets the stage for future endeavors, including the design and preparation of putative higher affinity UROD inhibitors *via* a combination of our *in silico* docking methods with 1R3Q and 1R3Y, synthesis, and enzymatic testing. Ongoing efforts are focused on incorporating structure activity relationship (SAR) studies, as well as the development of cellular assays for UROD activity to support further the enzymatic assays used in the current study.

The current data confirms the at-least-additive activity of UROD inhibition with radiation and cisplatin in FaDu cells, as previously observed with siUROD [1]. Potent synergy, however, was only observed in the case of cisplatin combination. Next-generation higher affinity UROD inhibitors may induce potent radiosensitization.

The present system and future UROD inhibitors will facilitate investigations into the use of UROD inhibition as a means of achieving control across a wide variety of cancers, with and without combination therapy. Inhibitors can be tested on other HNSCC lines, primary human HNSCC cells, with/without cisplatin, and with/without other therapeutics used in head and neck cancer treatment, such as carboplatin, 5-fluorouracil, and cetuximab [37]. Panels of cells from a variety of cancers can also be tested to identify the most effective cancer types for such further study.

In summary, PI-16 was designed based on known and proposed UROD interacting compounds, docked to human UROD structures 1R3Q and 1R3Y *in silico*, and validated to inhibit UROD biochemically. This 1^st^ generation UROD inhibitor reduced cancer cell viability, while having limited effects on normal cells. Moreover, it could be combined effectively with radiation and cisplatin. On this basis, we propose that the design and preparation of additional UROD inhibitors could have a role to play in the generation of yet-improved cancer therapies and radiation sensitizers.

## Supporting Information

Figure S1 **Alternative synthetic targets included nonconjugated porphyrin derivatives, such as porphodimethenes, oxophlorins, and sulfur-bridged macrocycles.** Over a year was dedicated to the requisite synthetic effort. However, only PI-16 proved sufficiently stable to allow for its analysis as a potential UROD inhibitor. The negative control porphyrin did not significantly inhibit UROD, as per our proposition that a non-oxidized tetrapyrrole is required for activity.
(TIF)

Figure S2 A) ^1^H NMR spectrum of compound **6** recorded in CDCl_3_ containing a small amount of TFA. B) ^13^C NMR spectrum of compound **6** recorded in CDCl_3_ containing a small amount of TFA. C) ^1^H NMR spectrum of PI-16 as recorded in TFA. D) High resolution mass spectrogram of PI-16.
(TIF)

Figure S3 A) Structure of PI-16 with specific pyrrole (P), oxygen (O) and hydrogen (H) atoms labelled to facilitate a description of the interactions with: B) UROD 1R3Q and C) UROD 1R3Y.
(TIF)

Figure S4 **Mouse xenograft model characterization of PI-16.** A) FaDu (2.5×10^5^) cells were injected into the left gastrocnemius muscle of SCID mice to establish xenograft tumors. When tumor-plus-leg-diameter reached 7.5 mm, mice were treated with 40 mg/kg PI-16 (or buffer control), IP daily×6 days, +/−2×2 Gy localized radiation therapy on days 2 and 5 (n = 3 mice/group). The time-to-endpoint (tumor-plus-leg diameter = 13.5 mm) was longer in PI-16 and PI-16+RT treated mice compared to respective controls. B) Body weights were tracked for 25 days with no significant toxicity observed. Arrows represent IP injection, “X” represents radiation.
(TIF)

File S1 **Supporting Information.**
(DOCX)
