# Supplementary figures and images for: A Porphodimethene Chemical Inhibitor of Uroporphyrinogen Decarboxylase
Source: PLoS One. 2014 Feb 25;9(2):e89889. doi: 10.1371/journal.pone.0089889 (PMC3934957; doi:10.1371/journal.pone.0089889)

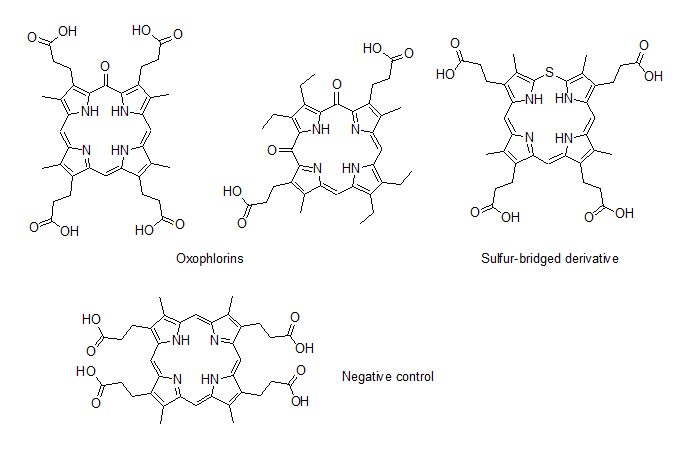

Supplement: Figure S1 — Alternative synthetic targets included nonconjugated porphyrin derivatives, such as porphodimethenes, oxophlorins, and sulfur-bridged macrocycles. Over a year was dedicated to the requisite synthetic effort. However, only PI-16 proved sufficiently stable to allow for its analysis as a potential UROD inhibitor. The negative control porphyrin did not significantly inhibit UROD, as per our proposition that a non-oxidized tetrapyrrole is required for activity. (TIF) [file pone.0089889.s001.tif]

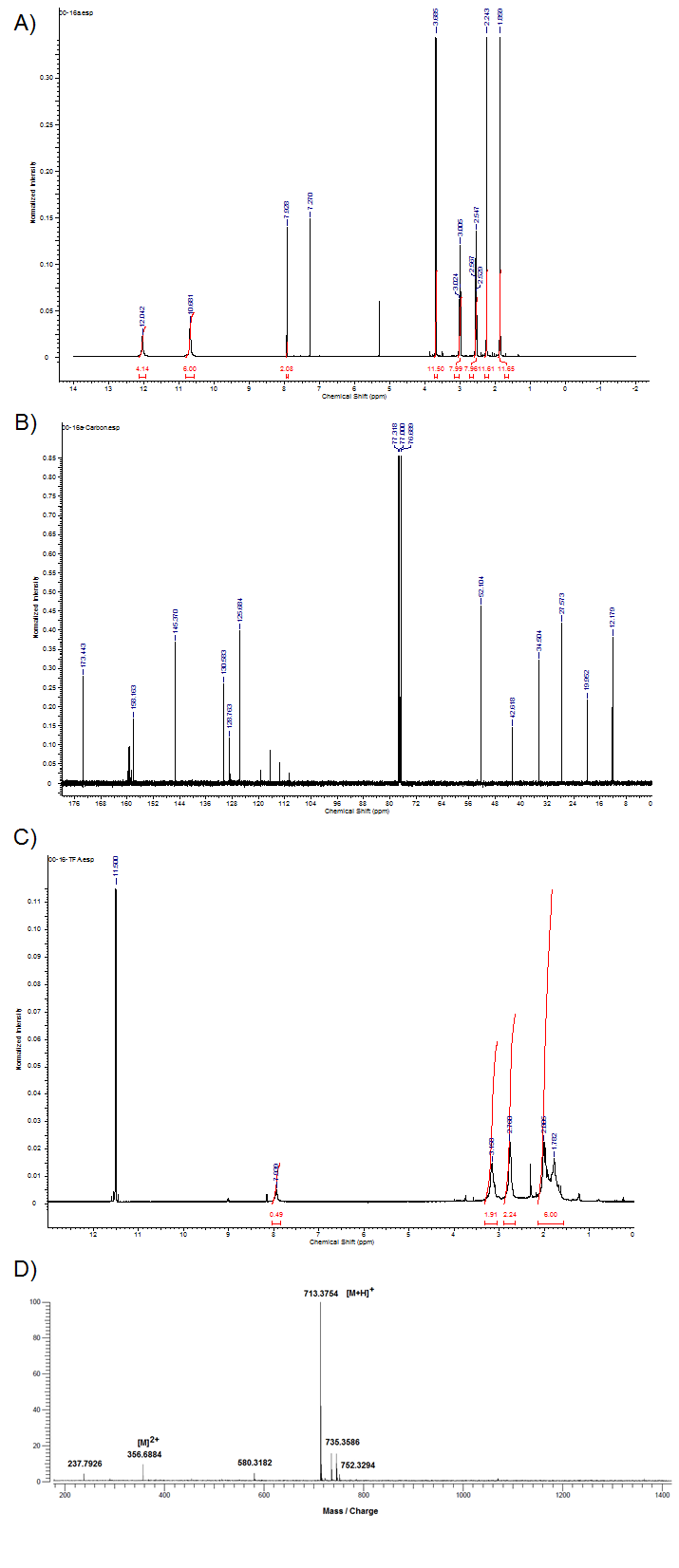

Supplement: Figure S2 — A) 1H NMR spectrum of compound 6 recorded in CDCl3 containing a small amount of TFA. B) 13C NMR spectrum of compound 6 recorded in CDCl3 containing a small amount of TFA. C) 1H NMR spectrum of PI-16 as recorded in TFA. D) High resolution mass spectrogram of PI-16. (TIF) [file pone.0089889.s002.tif]

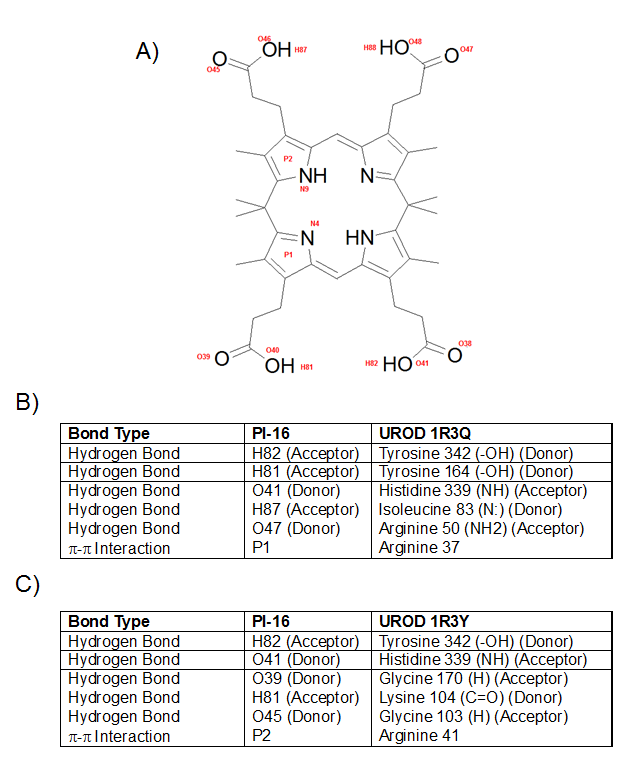

Supplement: Figure S3 — A) Structure of PI-16 with specific pyrrole (P), oxygen (O) and hydrogen (H) atoms labelled to facilitate a description of the interactions with: B) UROD 1R3Q and C) UROD 1R3Y. (TIF) [file pone.0089889.s003.tif]

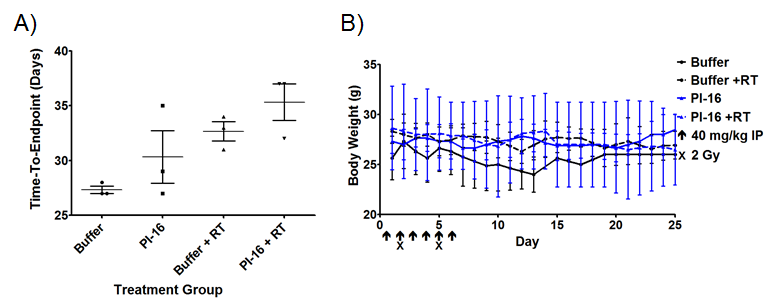

Supplement: Figure S4 — Mouse xenograft model characterization of PI-16. A) FaDu (2.5×10^5^) cells were injected into the left gastrocnemius muscle of SCID mice to establish xenograft tumors. When tumor-plus-leg-diameter reached 7.5 mm, mice were treated with 40 mg/kg PI-16 (or buffer control), IP daily×6 days, +/−2×2 Gy localized radiation therapy on days 2 and 5 (n = 3 mice/group). The time-to-endpoint (tumor-plus-leg diameter = 13.5 mm) was longer in PI-16 and PI-16+RT treated mice compared to respective controls. B) Body weights were tracked for 25 days with no significant toxicity observed. Arrows represent IP injection, “X” represents radiation. (TIF) [file pone.0089889.s004.tif]
