# Supplementary material for: A Porphodimethene Chemical Inhibitor of Uroporphyrinogen Decarboxylase
Source: PLoS One. 2014 Feb 25;9(2):e89889. doi: 10.1371/journal.pone.0089889 (PMC3934957; doi:10.1371/journal.pone.0089889)
Supplement: File S1 — Supporting Information. (DOCX) [file pone.0089889.s005.docx]

**File S1: Supplementary Information** - A Porphodimethene Chemical Inhibitor of Uroporphyrinogen Decarboxylase, Yip *et al.*

**Text S1. NMR/MS Spectra**

NMR spectra for compound **6** and PI-16 were recorded in CDCl_3_ with a small amount of TFA as described in the main text.

**Text S2. Mouse Xenograft Model Characterization of PI-16**

All animal experiments were conducted in accordance with the guidelines of the Animal Care Committee, Ontario Cancer Institute, University Health Network (Toronto, Canada). Further, all animal experiments used 6-8 week old severe combined immunodeficient (SCID) BALB/c female mice obtained from the Animal Research Colony, Ontario Cancer Institute. Briefly, 2.5 x 10^5^ FaDu cells were injected into the left gastrocnemius muscles of the mice, and tumors were allowed to form. Once the tumor-plus-leg-diameter reached 7.5 mm (approximately 2 weeks), the mice were randomized into the following treatment groups (n=3 mice/group): buffer, PI-16, buffer and radiation therapy, and PI-16 and radiation therapy. Buffer and PI-16 (40 mg/kg) were administered using intraperitoneal (IP) injections, once a day for 6 days. Localized radiation therapy was administered at 2 Gy/dose, on the 2^nd^ and 5^th^ day of treatment.
